# Leptin Regulated ILC2 Cell through the PI3K/AKT Pathway in Allergic Rhinitis

**DOI:** 10.1155/2020/4176082

**Published:** 2020-03-07

**Authors:** Qingxiang Zeng, Xi Luo, Yiquan Tang, Wenlong Liu, Renzhong Luo

**Affiliations:** Department of Otolaryngology, Guangzhou Women and Children's Medical Center, Guangzhou Medical University, Guangzhou, China

## Abstract

**Background:**

Recent studies suggest that leptin is involved in Th2 response in allergic rhinitis (AR). However, the effect of leptin on type II innate lymphoid cells (ILC2s) in AR is not well characterized.

**Methods:**

Twenty-six AR patients and 20 healthy controls were enrolled. Serum leptin levels were measured, and their correlation with ILC2 and type II cytokines were analyzed using enzyme-linked immunosorbent assay (ELISA) and flow cytometry. ILC2 differentiation and cytokine production stimulated by human recombinant leptin were analyzed by real-time polymerase chain reaction (PCR) and ELISA. AR mouse models were also established to verify the effect of leptin on ILC2 cell regulation.

**Results:**

Our results showed that elevated serum leptin in AR patients was correlated with the percentage of ILC2 and the expression of type II cytokines. The recombinant leptin enhanced the expression of ILC2 cell transcription factors and type II cytokine through the PI3K/AKT pathway. The AR mice treated with leptin showed as stronger ILC2 inflammation and symptoms compared with control mice.

**Conclusions:**

Our data provide evidence that upregulation of leptin promotes ILC2 responses in AR and this process was achieved through the PI3K/AKT pathway.

## 1. Background

Epidemiological survey shows that there are about 500 million allergic rhinitis (AR) patients worldwide, affecting the quality of life of 10%-20% of the population [1]. In recent years, the incidence of AR in China has been increasing year by year. It is worth noting that the incidence of obesity is also increasing synchronously [2]. Obesity has been proved to be an important risk factor for the occurrence and development of respiratory allergic inflammation (especially asthma) [3].

White adipose tissue can mediate metabolic effects by secreting a large number of adipose factors in obese patients, including leptin and adiponectin, in which leptin is considered to be the core medium linking nutrition, metabolism, and immune homeostasis [4]. Leptin has been proved to promote Th1 response in both humans and mice, but its role in Th2 response remains unclear [5–7]. Previous studies have shown that the serum levels of leptin in patients with AR are significantly higher and positively correlated with the severity of clinical symptoms [8, 9]. The combination of leptin and its receptor activates JAK2-STAT3, MAPK, and PI3K-AKT pathways [10, 11].

ILC2 is widely distributed in adipose-associated lymphoid tissue, intestine, lung, and skin and is an important member of the early stage of immune response. Allergens directly activate ILC2 by inducing the secretion of epithelial-derived cytokines such as IL-25, IL-33, and TSLP to produce IL-13, IL-5, IL-4, and IL-9 [12]. Studies had found that ILC2 in lungs of obese mice did not decrease despite that the number of ILC2 in visceral adipocytes of obese patients and mice decreased significantly [13, 14]. These results suggested that ILC2 was significantly affected under obese state. Studies also showed that the frequency of ILC2s was significantly decreased by high-fat-diet feeding and leptin deficiency-induced obesity [15].

In this study, we aimed to explore the effect of human recombinant leptin on the differentiation and function of ILC2 by both *in vivo* and *in vitro* studies.

## 2. Methods

### 2.1. Patients

Twenty-six AR patients without obesity and twenty healthy controls without obesity were recruited in this study. As described in Allergic Rhinitis and its Impact on Asthma guideline (2010), AR was diagnosed according to symptoms and duration, allergens test to common inhalant allergens (dust mites, pets, molds, cockroach, etc.) by skin prick test or specific IgE measurement [1]. The exclusion criteria included the following: atopic dermatitis, asthma, nasal anatomic abnormalities, and use of systemic corticosteroids in the previous 2 months. The study protocols were approved by local ethics committee boards, and written informed consent was obtained.

### 2.2. Flow Cytometry for ILC2

Peripheral blood mononuclear cells (PBMCs) were prepared using Lymphoprep (Fresenius Kabi Norge AS, Oslo, Norway) density-gradient centrifugation from heparinized leucocyte-enriched buffy coats. Isolated PBMCs were cultured at 2^∗^10^6^/mL in 24-well plates supplemented with RPMI-1640 with 5% human AB serum, 5 mmol/L glutamine, and penicillin and streptomycin solution (all from Invitrogen, except serum from Sigma-Aldrich). Then, PBMCs were stimulated by PMA (50 ng/mL) and ionomycin (500 ng/mL; both from Sigma-Aldrich) for 4 hours and by brefeldin A (BD, Oxford, United Kingdom) for the final 3 hours of culture.

PBMCs from donors were stained by antibody mixture (FITC lineage cocktail: CD2(RPA-2,10), CD3(OKT3), CD14(61D3), CD16(CB16), CD19(HIB19), CD56(TULY56), CD235a(HIR2), eBioscience, San Diego, CA) and FceRI (9E1, eBioscience) for the exclusion of B, T, natural killer, and natural killer T cells as well as mast cells and basophils. This process left approximately 50% pure Lin^−^ cells. The Lin^−^ cells were then stained with APC/Cy7-conjugated CD45 antibody (2D1), PE-conjugated CRTH2 antibody (BM16, BD Biosciences, NJ), and PE-Cy7-conjugated CD127 antibody (HIL-7R-M21, BD Biosciences, NJ) for the identification of human ILC2s. The human ILC2s were identified as Lin^−^ CD45^+^ CRTH2^+^CD127^+^ lymphocytes. The mice ILC2s in PBMCs were identified as Lin^−^ (TCR*β*(H57,597), TCR*γδ*(eBioGL3), CD3*ε*(145,2C11), Gr-1(RB6,8C5), CD11b(M1/70), TER-119(TER,119), B220(RA3,6B2), NK1.1(PK136), CD5(53,7.3), and CD11c(N418)) ST2^+^ (RMST2,2) CD45^+^(104) CD127^+^ (A7R34) lymphocytes. IL-5(JES1-39D10), IL-4(3010.211), and IL-13 (JES10-5A2) positive ILC2 cells in PBMCs were determined by intracellular cytokine staining using Cytofix (BD Biosciences, NJ) according to the manufacturer's instructions. The Beckman flow cytometer machine was used in the test (Beckman Coulter, Hercules, CA, USA).

### 2.3. ILC2 Sorting

Using the EasySep FITC selection kit, the peripheral blood lineage positive (Lin^+^) cells in PBMCs were removed, and then lineage negative (Lin^−^) cells were enriched. Then, the Lin^−^ CRTH2^+^CD127^+^ cells (1.5 × 10^5^ cells/mL) were enriched through staining with PE-labeled CRTH2 and PE-Cy7-labeled CD127 using a MoFlo XDP cell sorter (Beckman Coulter, CA, USA) and cultured in RPMI-1640 with 10% FBS and 1% penicillin/streptomycin for 3 days. The viability was larger than 98% (Trypan blue staining). At the same time, IL-25 (10 ng/mL), IL-33 (10 ng/mL), TSLP (10 ng/mL), and IL-2 (50 ng/mL) were added. For stimulation experiments, 100 ng/mL leptin, 100 ng/mL LY294002 (PI3K specific inhibitor), 100 ng/mL AG490 (JAK inhibitor), and 100 ng/mL SB203580 (MAPK inhibitor) were added. PBS was used as negative control. All cytokines and inhibitors were purchased from R&D Systems. A dose response experiment was performed to determine the concentration of stimulators. The stimulation concentrations of different stimulators were confirmed in pretest. The proliferation rate was assessed through incorporation of tritiated thymidine.

### 2.4. Quantitative Real-Time PCR (qRT-PCR)

The total RNA was isolated from ILC2s with TRIzol reagent (Life Technologies, Carlsbad, California). RNA (1 *μ*g) was digested by DNase I, extracted with phenol : chloroform (3 : 1), precipitated with ethanol, washed with ethanol, dissolved in RNAse-free water, and reverse-transcribed by the cDNA kit (Qiagen). PCR amplification was detected using an ABI PRISM 7300 Detection System. The data were calculated by the 2^-*ΔΔ*Ct^ method. The relative expression of target genes was normalized to GAPDH. The primers used in the test were listed as follows: GATA3 sense, 5′-GCGGGCTCTATCACAAAATGA-3′, antisense, 5′-GCTCTCCTGGCTGCAGACAGC-3′; ROR*α* sense, 5′-AAGGAGCCAGAAGGGATGAAC-3′, antisense, 5′-GGAACA ACAGACGCCAGTAAG-3′; GAPDH sense, 5′-AGCCACATCGCTCAGACAC-3′, antisense, 5′-GCCCAATACGACCAAATCC-3′.

### 2.5. Western Blot

ILC2 cells were lysed by cold RIPA buffer, and the samples were separated using 10% SDS-polyacrylamide gel electrophoresis (PAGE). After blocked by 5% TBST-milk for 60 minutes, PVDF membranes were incubated with mouse anti-pAKT (5.Ser 473) and *β*-actin (clone:C4) (Santa Cruz, 1 : 1000) overnight under 4°C. After incubation with horseradish peroxidase-labeled anti-rabbit IgG antibody, the signals were measured by the enhanced chemiluminescence kit (Pierce, Rockford, IL).

### 2.6. Enzyme-Linked Immunosorbent Assay (ELISA)

The concentration of type II cytokines (IL-4, IL-5, and IL-13) in the culture supernatant of ILC2 isolated from blood of patients and leptin levels in serum was detected by ELISA kits (R&D Systems, USA) as the protocol provided by the manufacturer. The sensitivity of the assays was as follows: IL-4, 0.22 pg/mL; IL-5, 3.9 pg/mL; IL-13, 125 pg/mL; and leptin, 15.6 pg/mL.

### 2.7. Animal Model

BALB/c mice were housed in the specific pathogen-free barrier facility with free access to food and water. On day 0 and day 7, the mice were given 100 *μ*L of PBS containing 100 *μ*g of OVA (1 *μ*g/*μ*L) and 1.6 mg Al(OH)_3_ intraperitoneally. The 10 *μ*g of OVA in 100 *μ*L of PBS with or without 10 *μ*g/mL leptin, 10 *μ*g/mL antileptin (neutralization antibody), or 10 *μ*g/mL LY294002 was given nasally on days 15, 16, and 19 with as previously described [16]. All cytokines and inhibitors were purchased from R&D System. The mice were killed the day after the last nasal stimulation. The frequency of ILC2 cells in PBMCs was determined by flow cytometry.

### 2.8. Statistical Analysis

All data are showed as mean ± SD. The Kruskal-Wallis H test or the nonparametric Mann-Whitney *U* test was performed except additional note. Correlations between leptin and other parameters were assessed by the Spearman rank correlation analysis. A *P* value of less than 0.05 was defined as statistically significant.

## 3. Results

### 3.1. The Serum Levels of Leptin and Its Correlation with ILC2 Frequency and Production of Type II Cytokines

**Figure 1:**
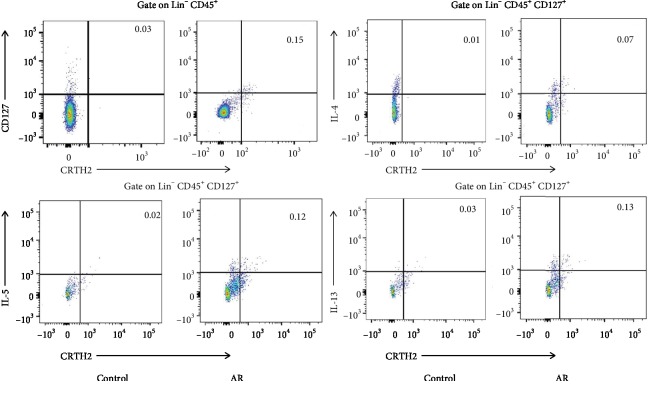
Representative flow cytometry showed elevated ILC2 (Lin^−^ CD45^+^CRTH2^+^CD127^+^) frequency, IL-4^+^ ILC2 frequency, IL-5^+^ ILC2 frequency, and IL-13^+^ ILC2 frequency in PBMCs from AR compared with controls. All the frequencies of ILC2s were converted into proportions in PBMCs (per mL of blood). Three independent tests were performed for every experiment.

**Figure 2:**
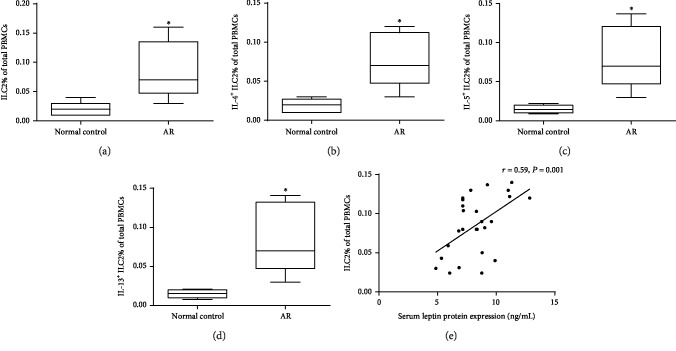
Flow cytometry confirmed elevated ILC2 frequency (a), IL-4^+^ ILC2 frequency (b), IL-5^+^ ILC2 frequency (c), and IL-13^+^ ILC2 frequency (d) in PBMCs from 26 AR compared with 20 controls. The correlation between serum levels of leptin and ILC2 frequency (Lin^−^ CD45^+^CRTH2^+^CD127^+^) was shown in (e). ^∗^*P* < 0.05. Three independent tests were performed for every experiment.

The information of participants is listed in Table 1. The serum levels of leptin in the AR group were significantly higher compared with control (9.8 ± 2.3 ng/mL *vs.*3.6 ± 1.5 ng/mL, *P* < 0.05). The frequency of ILC2, IL-4^+^ ILC2, IL-5^+^ ILC2, and IL-13^+^ ILC2 was significantly elevated in AR patients compared with controls (Figures 1 and 2). The elevated serum leptin levels were significantly correlated with the frequency of ILC2 (Figure 2) and the frequency of IL-4^+^ ILC2 (*r* = 0.46, *P* < 0.05), IL-5^+^ ILC2 (*r* = 0.53, *P* < 0.05), and IL-13^+^ ILC2 (*r* = 0.71, *P* < 0.05).

### 3.2. Expression of ILC2 Cell Transcription Factors and Type II Cytokine Stimulated by Leptin

**Figure 3:**
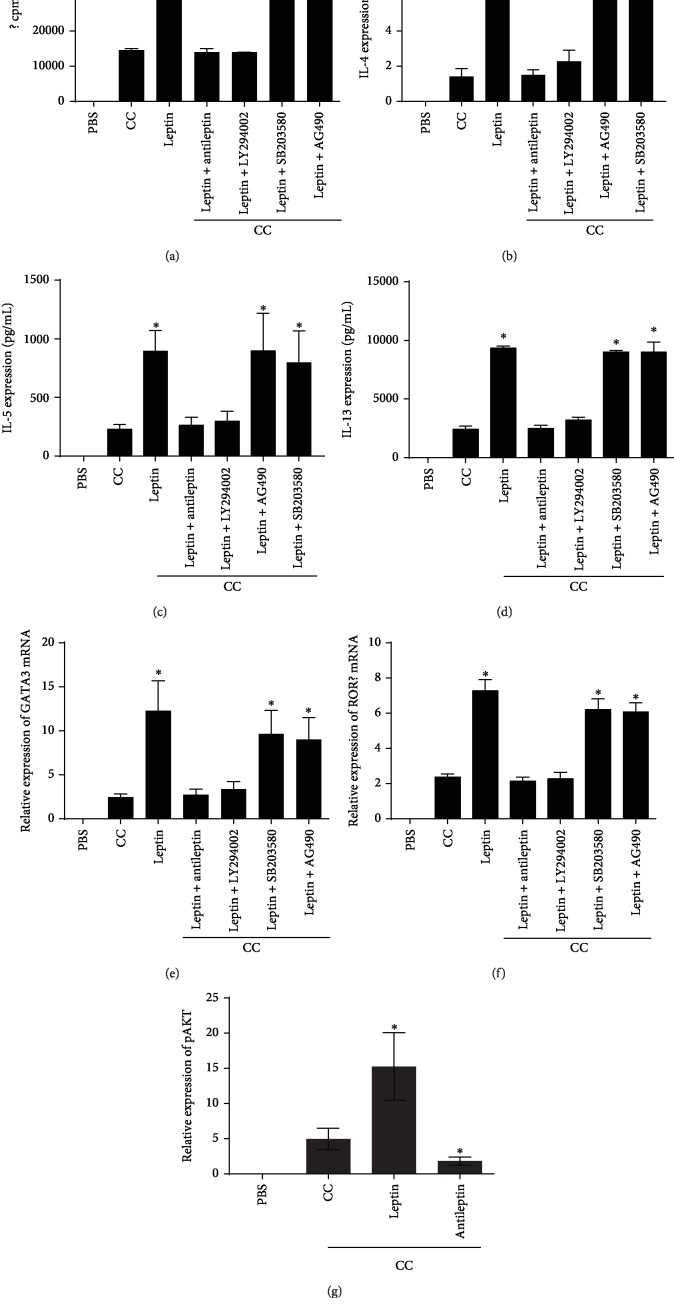
Proliferation of ILC2 was assessed by using tritiated thymidine incorporation under different stimulation shown in (a). The protein expression of IL-4, IL-5, and IL-13 in the culture supernatant of ILC2 isolated from blood of patients tested by ELISA and mRNA expression of GATA3 and ROR*α* in ILC2 isolated from blood of 10 patients tested by RT-PCR after stimulated by leptin (b–f). The AKT phosphorylation was tested with or without leptin treatment in ILC2s (f). ^∗^*P* < 0.05. LY294002, PI3K-specific inhibitor, AG490, JAK inhibitor, SB203580, and MAPK inhibitor. All the stimulators were 100 ng/mL, and stimulation time was 3 days. Cytokine combinations (CC) of IL-25 (10 ng/mL), IL-33 (10 ng/mL), TSLP (10 ng/mL), and IL-2 (50 ng/mL) were added in all the cultures. Three independent tests were performed for every experiment.

After stimulated by leptin, the expression of GATA3 and ROR*α* by ILC2 isolated from blood of patients was increased significantly. The type II cytokine production in the culture supernatant of ILC2 isolated from blood of patients was also upregulated after leptin stimulation. However, the above effect was inhibited when PI3K-AKT inhibitor or antileptin was added in the system (Figure 3).

### 3.3. Leptin Promotes ILC2 Responses in Mice

**Figure 4:**
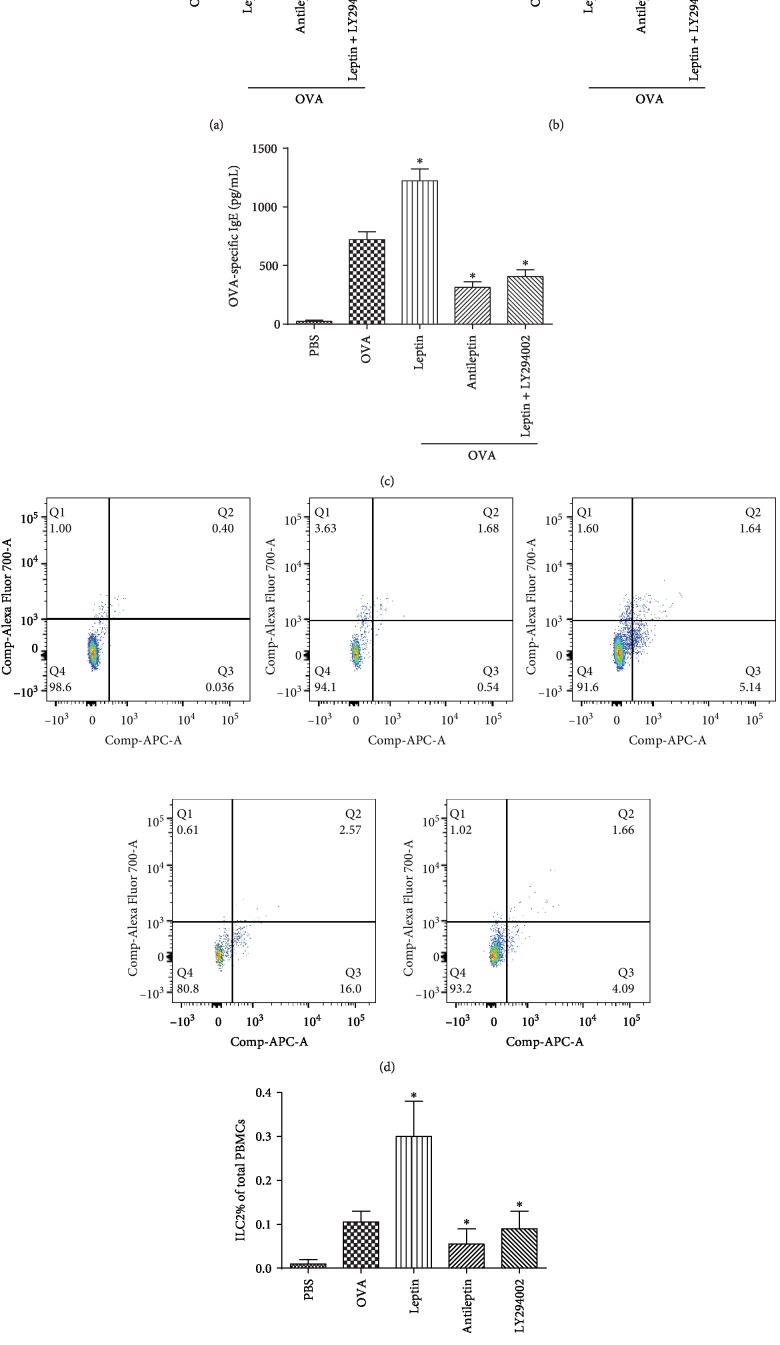
The times of nasal rubbing and sneezing (a, b), the serum OVA-specific IgE level determined by ELISA (c), and the frequency of ILC2 cells in PBMCs determined by flow cytometry in 30 AR mice (five mice in every group) treated with leptin or antileptin (d, e). All the stimulators were 100 ng/mL. All the frequencies of ILC2s were converted into proportions in PBMCs (per mL of blood). ^∗^*P* < 0.05. LY294002, PI3K-specific inhibitor. Three independent tests were performed for every experiment.

The serum leptin concentration, the level of OVA-specific IgE, the times of nasal rubbing and sneezing, and the frequency of ILC2 in PBMCs were significantly higher in AR mice than that of control mice, especially in the mice treated with leptin. Our results also showed that the antileptin attenuates allergic inflammation and the frequency of ILC2 (Figure 4).

## 4. Discussion

In this study, we explored the effects of leptin on ILC2 by cell and animal models. Our results confirmed that leptin can regulate the function of ILC2 by targeting the PI3K-AKT pathway.

Leptin is mainly secreted by the adipose tissue and also present in lymphoid organs [10, 11]. Since the obese patients were excluded, we believed that lymphoid organs may be an important source of leptin. Moreover, the effect of leptin tolerance was also avoided by excluding the obese subjects. Therefore, further studies with obese AR patients were needed to clarify the role of leptin tolerance in innate immunity.

In adaptive immunity, leptin is found to induce Th1 response in both humans and mice, but its effect in proallergic Th2 response remains controversial. Recent studies proved that leptin promoted Th2 cell proliferation, survival, and cytokine production. Moreover, our previous study showed that leptin also involved Th17 immune responses by inducing ROR*γ*t transcription in AR [6, 7, 17]. Our results also suggested that local treatment of leptin promotes systemic IgE production, which may be attributed to the effect of elevated local expression of IL-4 on bone marrow through blood circulation. Besides, the proliferation and cytokine production by Th2 cell promoted by leptin can also induce the expression of IL-4, which contribute to IgE production in both humans and mice. However, the role of leptin in innate immunity was not characterized well.

In the present study, we found that the serum levels of leptin were significantly higher in AR patients compared with controls and correlated with the level of type II cytokines. We also demonstrated that leptin, an adipokine elevated in obese individuals, promoted the function of ILC2, proved by upregulated expression of GATA3 and ROR*α* and type II cytokine production. Consistently, Zheng et al.'s study also showed that leptin promoted proliferation and survival of Th2 cells and ILC2 and production of type 2 cytokines, which together contribute to allergic asthma [18].

Leptin acts to regulate immune cell function by multiple signaling pathways, such as JAK2-STAT3, MAPK, and PI3K-AKT [10, 11]. For example, the leptin-STAT3 pathway has been proved to mediate cell survival in hippocampal neurons [19]. Moreover, leptin exerted an antiapoptotic effect by activating ERK1/2 and AKT-mTOR pathways in Th1/Th17 cells [20]. Interestingly, the antiapoptotic effects of leptin are dependent on MAPK activation, rather than the PI3K pathway in human Jurkat T cells [21]. Our present study showed that leptin mediated the effect of ILC2 through the PI3K-AKT pathway in AR. Our *in vivo* study also suggested that leptin promoted ILC2 inflammation through the PI3K-AKT pathway, which confirmed the *in vitro* results.

There are also some limitations in our study. First of all, the sample size is small and further studies with large sample size are needed. Secondly, the obese subject in our study was excluded to eliminate the effect of obese state on the expression of leptin. Therefore, the study of leptin on the regulation of ILC2 in obese AR patients was needed. Thirdly, further studies with Rag-deficient mice lack of lymphocytes should be used to confirm the effects of leptin in ILC2s. Fourthly, systemic treatment of leptin in mouse model should be studied to explore the systemic effect of leptin on the regulation of ILC2.

## 5. Conclusions

In summary, our findings suggested leptin-mediated regulation of the differentiation and function of ILC2 cells through leptin and PI3K-AKT signaling pathway, providing a new potential treatment target.

## Figures and Tables

**Table 1:** Demographic characteristic of AR and control patients.

Groups	AR group	Control
Number	26	20
Sex (male : female)	14 : 12	11 : 9
Age (years)	29.8 (18-51)	32.4 (21-56)
BMI	20.3 (18.9-22.4)	21.6 (19.2-23.1)
ECP (ng/mL)	61.3 (22.6-127.3)^∗^	18.1 (7.5-98.2)
TIgE (IU/mL)	148.9 (37.2-638.2)^∗^	31.5 (9.1-56.7)

^∗^Compared with the control group, *P* < 0.05.

## Data Availability

The datasets used and/or analyzed during the current study are available from the corresponding author on reasonable request.

## Abbreviations

AR: Allergic rhinitis
ILC2: Type II innate lymphoid cells
ELISA: Enzyme-linked immunosorbent assay
qRT-PCR: Quantitative real-time PCR.
